# The Ebola contagion and forecasting virus: evidence from four African countries

**DOI:** 10.1186/s13561-015-0047-5

**Published:** 2015-06-14

**Authors:** Selmi Nadhem, Hachicha D Nejib

**Affiliations:** Department of Quantitative Methods, Faculty of Economics and Management of Sfax, Sfax, Tunisia

**Keywords:** Ebola 2014, Contagion, Virus, EGARCH, MGARCH-DCC

## Abstract

This paper is focused on examining the number of deaths’ increases participation in the propagating the Ebola virus during the period ranging from March to October 2014. An application of the MGARCH-DCC model regressions on four countries has led to discover that the finding that human contact play a significant role in transmitting the Ebola virus. Our findings also reveal that Guinea has already suffered from a spread-like virus originating from Sierra Lione and Liberia. Noteworthy also, other countries are now liable to such a risk; for instance, Nigeria is a country vulnerable to the propagation of this virus. Consequently, we undertake to conduct our forecasts for EGARCH model estimates implements; which has estimated a decrease in the Ebola virus incurred number of deadly Ebola virus over the two months following the November and December.

## Background

The Ebola virus belongs to a family of viruses called filovirus^a^, commonly carrying the Marburg virus. Discovered in 1976 in the western equatorial province of Sudan and in a nearby region of northern Zaire (now Democratic Republic of Congo (DRC)) [1,2], the Ebola virus had affected several African countries between 1976 and 2008 (Centers for Disease Control and Prevention, 2014; [3,5]; Leroy et al., 2004; [6-12] and [13]. Previous Ebola virus modeling papers have examined its 1995 outbreak in Kikwit, DRC [14-16] and [17,18]), and the in Uganda in 2000–2001 (see [16,19] and [17]).

Currently, however, the Ebola has also had certain implications in Guinea, Sierra Leone and Liberia. Indeed, a small number of cases are reported in Nigeria (21 deaths) and a single case in Senegal (travelers arriving from Guinea). This consists, actually, in is the contagious Ebola virus spread among countries. Until September 8, 2014, 4366 cases had been reported, including 2218 deaths. The Ebola virus is discovered to be the most dangerous epidemic, recently resulting in a remark able increasing in the number of patients and deaths it’s since the outbreak out of this virus. The first Ebola outbreak among human beings occurred in 1976 [20], with a spread contagion due mainly to travelling. An infected person travelled for instance from Guinea to Liberia may be carrying the infection, which would in turn infect some other people there (The institute of tropical medicine, 2013 [21]).

Based on the Ministry Health reported information (September 2014), Guinea recorded 1040 confirmed deaths highly exceeding the confirmed cases. Figures relevant to the Ebola cases and deaths have been provided by WHO, with the most prominent number being registered in Liberia, (more than 1760), while, 2450 probable deaths have been reported with respect to Sierra Leone. These data are based on official information reported by the Ministry of Health up the October 5th for both Guinea and Sierra Leone and October 4th for Liberia.

Concerning Guinea, one might well note that the number of confirmed cases is important in respect of the probable and suspected cases. Still, the Liberian probable cases remain the most significant in relation to the entirety of: confirmed and suspected cases. As for Sierra Lione confirmed cases, they constitute the highest proportion as compared to the other three remaining countries. Finally, Nigeria exhibits the weakest level of cases regarding the three indicators involving confirmed, probable and suspected cases.

Actually, five species of Ebola have been detected to prevail in Bundibugyo, Côte d’Ivoire, Reston^b^ [22], Sudan and Zaire. The outbreaks of Ebola virus associated of hemorrhagic fever has taken place mainly in Africa, with a death rate comprised 25% and 90%.

As a matter of fact, the Ebola virus is transmitted to human beings from wild animals, and then spreads from human-to-human. The 2014 Ebola epidemic is the most prominent in history, affecting aerial several countries. Since the beginning of 2014, some cases of Ebola virus infection have been reported in several sub-Saharan African countries. These countries, health authority’s line with the World Health Organization (WHO) and its different partners, have all been mobilized to prevent the virus transmission to other countries. Travelers to or from these countries can find relevant health care related information on the Ministry of Foreign Affairs and International Development website.

In effect, Ebola is a communicative disease, as it can be transmitted by either contact with blood, body fluids or tissues of infected subjects, or sick or dead animals. So for airborne transmission has been indicated to occur. The incubation period (the time between the infection and symptoms’ onset) ranges from 2 to 21 days. A person who bears no symptoms is not contagious. Most often, the risk degree of the Ebola virus contamination is considered too low during the disease the early stages but contagion of the Ebola virus makes the symptoms worsen or improve. People are infectious as long as their blood and secretions bear traits of the virus.

The outbreak of the Ebola virus tends to occur at irregular intervals in a medical care environment. The first trigger was reported in the Forest region of Guinea. The Ebola outbreaks in Liberia, Sierra Leone and Nigeria were contained [23]. Until recently, the Ebola virus has been raging mainly in central and eastern Africa. Contagion occurred between 1976 and 2012, in the Democratic Republic of Congo and Sudan along with the three other countries which had remarkably been affected by serious epidemics’, namely; Gabon, Uganda and Congo. Although Ebola has resulted in a high mortality rate, it’s the real varies between 25% and 90%, depending on the strain. According to the WHO (October 2014), Ebola currently stands within an average of 54% of total deaths [13].

**Figure 1:**
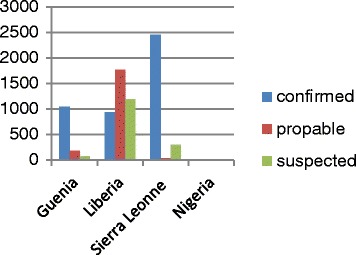
**Confirmed probable, and suspected of Ebola virus cases.**

Based on Figure 1, and for an effective combating of the epidemics some conditions and resources need be considered and ensured, namely case management, contact tracing and monitoring, laboratory quality, safe burials along with social mobilization. The community is involved in the outbreak control as early-stage awareness of the Ebola virus infection and the possible protective measures are likely to be successful. In fact, they have greatly helped in reducing contamination among humans. Messages about risk reduction should rather focus on social mobilization and community-based awareness campaigns to help reduce the disease propagation risk [24].

Indeed, the WHO intends to reduce the Ebola virus propagation risk between animals and human beings, about all contact with monkeys. In fact animals should be handled with gloves as well as other appropriate protective clothes. More importantly, animal resource based foods must be thoroughly cooked before eating them.

Other various factors may well contribute greatly in increasing or decreasing contamination risk [25]. In this respect, the present study attempts to provide certain suggestions whereby contamination risk to human beings can be reduced, a risk occurring mainly through direct contact with similar subjects, and, predominately with their body fluids. Normally, people need to wear gloves and appropriate personal protective equipment when taking care of an affected patient at home. To prevent such a contagion, people also have to wash out hands after visiting patients in hospitals. The various measures necessary for containing the Ebola outbreak include corpses ‘rapid burial and identifying subjects likely to be in contact with an infected person.

Concerning fight against infection in health care facilities, health workers should always apply the standard precautions on taking care of patients, regardless of the presumed diagnosis. These precautions pertain to the basic rules regarding hygiene including, wearing personal protective equipment (to protect oneself against splashing or direct contact with infected materials), safety injections as well as funeral rites.

Health workers responsible for taking care of the Ebola virus infection suspected or confirmed cases must avoid any contact with the patient’s blood or body fluids and with such contaminated surfaces as clothing and bed linen materials. On standing too close too sick person (say, i.e. within a distance of less than a meter), one has to should wear face protection (face shield or surgical mask and goggles), a clean sterile gown and gloves (sterile medical procedures). In him laboratory staff might well face the same risk. The Ebola samples taken from human beings or animals should be handled by specialized trained staff and processed in suitable equipped laboratories.

The WHO aims to prevent Ebola outbreaks by providing disease control and helping countries at-risk to develop special safe guarding measures and preparing plans. In this context, this present paper is dedicated to provide some general guidance regarding the fight against outbreaks caused by these two viruses. Both of the Ebola disease and Marburg virus outbreak and curbing stages are: preparation, warning, control and evaluation. Whenever an outbreak is detected, the WHO is involved in providing assistance through monitoring, supporting patients, mobilizing laboratory services, research contacts, infection control, logistic support training and assistance with safe burial practices. In This regards, the WHO has developed detailed preventive information to curb prevent and fight against with Ebola virus contamination.

Since December 2013, an Ebola virus originating hemorrhagic fever epidemic has predominantly prevailed in West Africa (Guinea, Liberia, Sierra Leone, and Nigeria are the major affected countries in addition to a confirmed case in Senegal). For the first time, such in West African countries as Congo, DRC and Uganda have been affected by with the Ebola virus. On the interest, international SOS organization is monitoring the situation closely and updates the site with the latest available information. In this respect, the present work constitutes a case study concerning of the Ebola virus transmission among the most affected countries by the virus (Guinea, Sierra Leone, Liberia and Nigeria).

This paper is designed to check whether these countries can be actually constitute a source of recent contagion judging by the number of Ebola virus incurred deaths. More particularly, this work is focused on showing whether the Ebola virus can produce contagious effects within the studied countries’ in conditional volatilities over the period ranging between March and October 2014. Previous contagion related studies have somewhat failed to take account for the important distinction between the concepts of interdependence and contagion^c^. Indeed, the conditional test results’ dynamic correlation reveal well that the contagion effects appear to be multidirectional since the Ebola virus emanating return shocks can actually sweeping across the entirely of the markets. Yet, the contagion in-volatility effects are mostly driven by the Ebola virus originating negative return shocks. Such an empirical finding reveals well that the number of death shocks can turn out to be contagious not only at the volatility level, but also at the mean level, indicating that Ebola outbreak can be a major source of contagion over the period March-October 2014.

In fact, the objective of the paper lies in highlighting that deaths’ number does actually constitute a contagion channel. In a first step, this study is concerned to test the persistence of shocks and the stylized facts of these countries’ considered Deaths returns number through EGARCH process [4]. In effect, the presence of structural changes in the series of conditional variances as detected via the ICSS algorithm heave motivated us to study Conditional Correlation Dynamics [26] in a second stage as a procedure to determine the contagion effect across markets. Finally, this study serves to help predict the number of Deaths’ volatility across the EGARCH process.

In this study, the cited process involves four major steps. On a first stage, the Iterated Cumulative Sums of Squares algorithm (ICSS) of [27] is applied to detect the presence of structural breaks in deaths number relevant to the studied countries. On a second stage, and structural breaks and asymmetry to be taken into estimation, the study undertakes to analyze the univariate EGARCH model and bring the structural breaks’ dummy variables into variance equation. Compared to the standard specification, the EGARCH framework exhibits several advantages namely: no need is imposed to artificially implement a non-negative constraint on the model parameters, while asymmetries are allowed under the EGARCH formulation. Dubbed dynamic conditional correlations such a methodology has actually been developed by [26]. In reality, the major advantage lying implementing the DCC-MGARCH models consist in detecting of plausible changes noticeable in the relationships governing the variables remains prevalent in the underlying studied data.

The remainder of this paper is structured as follow. Section Methods is devoted to highflying the surveyed econometric methodology. The relevant data and major empirical results are discussed in Section Empirical results. As for the ultimate section it comprises provides the pertinent concluding remarks, along with the retained economic and political implications.

## Methods

For the purpose of the determining whether the number of deaths’ volatility dynamics does actually differ from that of the countries, we, firstly, undertakes to expose the exact local Whittle and structural break tests, and, then, discuss the GARCH specifications so as to be able to capture the possible conditional dynamic dependencies likely to be noted in the number of death return volatilities.

### The Exact Local Whittle method

The classes of semi-parametric frequency domain estimators follow the local Whittle approach as suggested by [28] and analyzed by [29] (dubbing it Gaussian semi-parametric estimator). The analysis applied process is the following:

2.1 $$ {y}_t=\mu +{\displaystyle \sum_{j=0}^{t-1}{\varphi}_{j,d}{x}_{t-j}},t=1\dots T $$

As for the Local Whittle estimator, it defined as the maximization of the local Whittle likelihood purpose, such as:

2.2 $$ Q\left(g,d\right)= \log \left\{\frac{1}{m}{\displaystyle \sum_{j=1}^m{\lambda}_j^{2d}{I}_y\left({\lambda}_j\right)}\right\}-\frac{2d}{m}{\displaystyle \sum_{j=1}^m\left[ \log \left({\lambda}_j\right)\right]} $$

where: *m* = *m*(*T*) denotes a bandwidth number tending to infinity *T* → ∞ except at a slower speed than *T*;$I\left(\lambda \right)=\frac{1}{2\pi T}{\left|{\displaystyle \sum_{t=1}^T{e}^{it\lambda }}\right|}^2$, represents the periodogram of *X*_*t*_, and g_*x*_(λ) the spectral density of *X*_*t*_, ${\lambda}_j=\frac{2\pi j}{n}$, and *j* = 1,…,*n*.

A notable disadvantage as compared to log-periodogram estimation is that a statistical optimization is highly. Still, this estimator underlying assumptions are weaker than those pertaining to the log-periodogram regression (LPR) estimator. In this regard, [29] have show that while $d\in \left(-\frac{1}{2},\frac{1}{2}\right)$;

2.3 $$ \sqrt{m}\left({\widehat{d}}_{LW}-d\right)\overset{d}{\to }N\left(0,1/4\right) $$

Hence, the asymptotic distribution turns about to be extremely simple, which facilitates easy asymptotic inference. More particularly, this estimator is discovered to be more efficient than the LPR one. The reliability and asymptotic normality ranges concerning the Local Whittle estimator have explicitly been demonstrated by [30] and [31] to equate those associated the LPR estimator.

This exact LW procedure as frequency labelled, implies replacing ${\lambda}_j^{2d}I\left({\lambda}_j\right)$ in (2.1) by ${I}_{\Delta^{d_y}}\left({\lambda}_j\right)$, and is only valid if μ = 0 in (2.2). Since the relevant means are different from zero, [32] suggests demeaning {*y*_*t*_} with an appropriate estimator $\widehat{\mu}$, and computing the exact LW estimator starting from the demeaned data. So the objective function to be minimized turns out to be:

2.4 $$ {R}_E\left(m,d\right)= \log \left\{\frac{1}{m}{\displaystyle \sum_{j=1}^m{I}_{\Delta^d\left(y-\widehat{\mu}\right)}\left({\lambda}_j\right)}\right\}-\frac{2d}{m}{\displaystyle \sum_{j=1}^m \log \left({\lambda}_j\right)} $$

Where: ${I}_{\Delta^d\left(y-\widehat{\mu}\right)}\left({\lambda}_j\right)$ is the periodogram of ${\Delta}^d\left(y-\widehat{\mu}\right)$. For fractional differences, to be determined, it is assumed that {*y*_*t*_} is given by a process similar to equation (2.1). It turns out that the first sample observation *y*_1_ is a reliable mean estimator in the case of large values of *d*, while the usual arithmetic mean *ӯ* helps ensure a significant task for small coefficient values of *d*. In this way, [32] suggests putting forward the subsequent weighted estimator, such as:

2.5 $$ \widehat{\mu}(d)=v(d)\overline{y}+\left(1-v(d)\right){y}_1 $$

2.6 $$ v(d)=\left\{\begin{array}{l}1,\kern0.5em d\le 0.5\\ {}\frac{1+ \cos \left(4\pi d\right)}{2},\ 0.5<\mathrm{d}<0.75\\ {}0\kern1em \mathrm{d}\ge 0.75\end{array}\right. $$

For the purpose of attaining, a feasible procedure, he considers two necessary steps, the first of which serves to determine an estimator of $\widehat{d}$ independent from μ in order to get an estimator of the constant: $\widehat{\mu}=\widehat{\mu}\left(\widehat{d}\right)$. As for the second step, the slope and Hessian of *R*_*E*_(*m*,*d*) are used to compute the feasible estimator as follows:

2.7 $$ {\widehat{d}}_{2ELW}=\widehat{d}-\frac{R_E^{\prime}\left(m,\widehat{d}\right)}{R_E^{{\prime\prime}}\left(m,\widehat{d}\right)} $$

Besides, [32] demonstrates shows that the two-step ELW estimator (2ELW) proves to be consistent registering the same limiting distribution as the LW and ELW estimators under −0.5 < *d* < 2. Similarly, and as indicated as shown by [33], if an unknown mean (initial value) appears to undergo certain change by its sample average, simulations suggest that the ELW estimator is inconsistent for *d* > 1. It is actually for this reason that we undertake to apply the 2ELW. In addition, [33] resort to modify the ELW objective function in a bid to estimate the mean by means of combining two estimators: the sample average and the first observation. He indicates the resulting estimator as being a two Stage Exact Local Whittle (2ELW). Applying the tapered estimator of [30] in its first stage, the 2ELW estimator, bears the same $N\left(0,\frac{1}{4}\right)$ limit distribution for $N\left(-\frac{1}{2},2\right)$ and is consistent when $d>\frac{1}{2}$. Furthermore, the 2ELW estimator finite sample performance appears to inherit the 2ELW estimator, desirable properties. Moreover, it can also be computed with prior data de-trending (2ELWd) as in [33].

### Structural breakpoints detection

As developed by [27] the ICSS algorithm has been applied to detect the structural breakpoints on 4 series over the study period. As a starting point, the stock return for market *i* on day *t* can be written as:

2.8 $$ {r}_{i,t}=\left( \log {P}_{it}- \log {P}_{it-1}\right)\times 100 $$

where: (*P*_*i,t*_) is the closing number of deaths:

Next, we proceed by defining

2.9 $$ {a}_{i,t}={r}_{i,t}-{\mu}_i $$

where {*a*_*i,t*_} is with zero mean and unconditional variance ${\sigma}_t^2$, μ_*i*_ denotes country i average return. Let ${C}_k={\displaystyle \sum_{t=1}^k{a}_t^2},k=1,\dots, T$ be the cumulative sums of squares of {*a*_*t*_} series, then *D*_*k*_ statistic can be calculated as follows:

2.10 $$ {D}_k=\left(\frac{C_k}{C_T}\right)-\frac{k}{T},k=1,....,T and\ {D}_0={D}_T=0 $$

The ICSS algorithm is adopted to detect the multiple breaks in the unconditional variance of {*a*_*i,t*_} series. Thus, the *D*_*k*_ statistics based ICSS algorithm would initiate with testing the structural breaks over the whole sample. In doing so, the ICSS helps depict any significant break, by applying the new statistic to examine the break for each of the two sub-samples (defined by the break). The algorithm proceeds in such a way as the statistics become insignificant for the entirety of sub-samples defined by any significant break. On the last stage, a dummy variables set is set up for the normalized return volatility to be captured.

This section is devoted to provide a through description of the wavelet transform as applied to the number of the death data decompositions, together with the multivariate GARCH model as used in our proper analysis.

As countries co-movement is but an outcome of transmissions emitted from each country, the global country transmission is most often represented by the number of deaths occurring in transferred to other countries. For the sake of accounting for such interdependencies, we consider appealing to an MGARCH model including the EGARCH structural changes associated with the variances introduced by [4]. In what follows is a presentation of the dynamic model EGARCH (1, 1).

### The EGARCH model

The above GARCH specification helps restrict any shock effect on the conditional variance to be symmetric and within the same size, be it positive or negative. Noteworthy however, such a shock effect should disappear geometrically over time. Still it well-become a known fact notably with the help of death data, the different country reactions in accordance with to the shock size and sign. To overcome such limitations, several asymmetrical GARCH models, such as the Exponential GARCH model [34], have been introduced in this respect. Formally, an EGARCH (1, 1) model corresponds to the following:

2.11 $$ \ln \left({h}_t\right)={\delta}_0+{\delta}_1 \ln \left({h}_{t-1}\right)+{\gamma}_0\frac{\varepsilon_{t-1}}{h_{t-1}}+{\gamma}_1\frac{\left|{\varepsilon}_{t-1}\right|}{h_{t-1}} $$

With an EGARCH specification, positivity constraints on parameters are no longer required. Furthermore, it is henceforth possible to apprehend asymmetry in volatility reaction toward external shocks. Indeed, if γ_0_ > 0 (respectively γ_0_ < 0), a positive shock on the lagged conditional variance implies an increase (respectively a decrease) in current volatility.

Parameter γ_1_ helps capture the asymmetry effect associated with the shock size $\frac{\left|{\varepsilon}_{t-1}\right|}{h_{t-1}}$. If γ_1_ = 0, a positive innovation would them have the same effect on conditional variance as would a negative innovation, while for γ_1_ > 0, a high size shock should have more effect on conditional variance than should a low size shock. So for the Ebola virus contagion to be determined, the MGARCH-DCC model will be applied so that the virus propagation effect can be determined concerning four African countries (Guinea, Sierra Leone, Liberia and Nigeria).

### The multivariate GARCH -DCC model

In this section, the dynamic conditional correlations’ two-stage model will be treated as proposed by [26]. Let us for instance, consider a vector consisting of any two variables *Y*_*t*_ = [*y*_1*t*_ *y*_2*t*_]*’*. Each variable constitutes a constant function along with its own past values. Thus, the autoregressive process reduced form is written as:

2.12 $$ A(L){Y}_t=c+{\varepsilon}_t\ \mathrm{avec}\ {\varepsilon}_t\to N\left(0,{H}_t\right),\forall t=1,2,\dots, T $$

where: *A*(*L*) is the polynomial delay, and ε_*t*_ = [ε_1*t*_ε_2*t*_]*’*is a vector of residuals training from the estimation auto regression process for each variable whose variance-covariance matrix is described by *H*_*t*_ = {*h*_*i*_}_*t*_ with s.

Actually, the DCC-MGARCH model can be easily apprehended by rewriting the matrix of variance-covariance *H*_*t*_ such as:

$$ {H}_t={D}_t{R}_t{D}_t $$

where: ${D}_t= diag\left\{\sqrt{h_{it}}\right\}$ is the standard deviations diagonal matrix, a variable temporally different from the two previous equations’ estimation with a univariate GARCH process; *R*_*t*_ = {ρ_*ij*,*t*_} representing the conditional correlation coefficients matrix. The *D*_*t*_ contained elements are generated into a GARCH (P,Q) process, which can be formulated as:

2.13 $$ {h}_{it}={w}_i+{\displaystyle \sum_{p=1}^P{\alpha}_{ip}{\varepsilon}_{it-p}^2}+{\displaystyle \sum_{q=1}^Q{\beta}_{iq}{h}_{{}_{it-q}}} $$

In addition, [26] considers adopting a GARCH-type structure while modelling the correlations’ dynamics. Thus, an (M, N) order DCC process can be described by:

$$ {R}_t={\left({Q^{*}}_t\right)}^{-1}{Q}_t{\left({Q^{*}}_t\right)}^{-1} $$

2.14 $$ {Q}_t=\left(1-{\displaystyle \sum_{m=1}^M{a}_m-{\displaystyle \sum_{n=1}^N{b}_n}}\right)\overline{Q}+{\displaystyle \sum_{m=1}^M}{a}_m\left({\xi}_{t-m}{\xi}_{t-m}^{\hbox{'}}\right)+{\displaystyle \sum_{n=1}^N{b}_n}{Q}_{t-n} $$

where: ${\xi}_t=\left\{{\varepsilon}_{it}/\sqrt{h_{it}}\right\}$ is the vector englobing the standardized residuals derived from the univariate GARCH model estimation, as a matrix of these standardized residuals’ conditional variance-covariance, whereas *Q*_*t*_ = {*q*_*ij,t*_} represents the unconditional variance-covariance matrix, which are temporally invariant. The parameters (*a*_*m*_;*b*_*n*_) are supposed to respectively intercept the shock effects and delay the dynamic correlations at the level of recent contemporary. As for *Q* ^*^_t_, it stands for a diagonal matrix containing the square root of the main diagonal elements of *Q*_*t*_. Regarding to our example, this matrix is written as:

2.15 $$ {Q^{*}}_t=\left(\begin{array}{l}\sqrt{q_{11}}\\ {}0\kern1.5em \sqrt{q_{22}}\end{array}\right) $$

with: ${\rho_{12,}}_t=\frac{q_{{{}_{12,}}_t}}{\sqrt{q_{{{}_{11,}}_t}{q}_{{{}_{22,}}_t}}}$ denoting the dynamic conditional correlations i.e. the matrix elements *R*_*t*_ whose main diagonal consists of 1.

The model parameters are estimated via the maximum likelihood DCC method. In this regard, [35] have show that the log-likelihood function can be expressed as:

2.16 $$ L=-\frac{1}{2}{\displaystyle \sum_{t=1}^T\left\{2 \log \left(2\pi \right)+2 \log \left|{D}_t\right|+ \log \left|{R}_t\right|+{\xi}_t{R}_t^{-1}{\xi}_t\right\}} $$

The estimation process involves two steps, the first of which consists in substituting an identity matrix by a matrix *R*_*t*_ within the log-likelihood the function. The advantage of such a method is that it allows for getting the likelihood function sum of the GARCH univariate models. In other words, this initial step, series to achieve we obtain the parameters of equation (2.13). As for the second step, it consists in estimating the equation (2.14) parameters via adopting the original likelihood function as described by equation (2.16). This procedure ensures maintaining the dynamic correlations among the studied variables.

## Results

### Data and descriptive statistics

The empirical data comprise total number of daily^d^ deaths caused by the Ebola virus as calculated by “OMS” of four African countries. Actually, the number of deaths recorded exclusively in Guinea, Sierra Leone, Liberia and Nigeria has been selected as subject of study as these four countries are the most affected with the Ebola virus. The sample ranges over the period comprised between March 2014 and the end of October 2014^e^, yielding 206 observations for each series.

Table 1 presents a wide range of descriptive statistics concerning the four series under investigation during the period (March-October 2014). The null hypothesis of no ARCH effects is rejected at a significance level of 1%, suggesting that the GARCH parameterization might well fit for the conditional variance processes.

**Table 1:** **Summary of descriptive statistics**

	**Guinea**	**Sierra Leone**	**Liberia**	**Nigeria**
T	206	206	206	206
Moy	3.951	5.742	11.932	0.038
Var	40.085	309.694	565.89	0.0037
T-stat	8.957*	4.683*	7.199*	2.877*
Skew	4.164*	7.951*	3.283*	4.809*
Kurt	22.687*	73.768*	16.933*	21.333*
J-B	5013.42*	48879.33*	2831.32*	4700.51*
ARCH	21.54*	9.35*	8.76*	14.39*
LB(24)	43.25*	38.807*	45.48**	37.33*

Notes: *indicate the significance level at 5%. **indicate the significance level at 1%.

In Table 1, the sample size and the unconditional mean indicate that the number of average daily deaths is fixed at 3.9 for Guinea, 5.7 for Sierra Leone, 11.9 for Liberia and 0.03 for Nigeria. With respect to Liberia and Sierra Leone, the sample provides a higher average return with a higher unconditional variance, highlighting the prevalence of a strong dispersion around the Ebola virus incurred deaths in these two countries. It is actually, this high dispersion feature which provides us with significant breaks through the conditional variance. Furthermore, distribution of the number of the virus related deaths seems to be symmetrical and leptokurtic, implying rejection of the normality null hypothesis. The mean and variance will, conditionally, be modelled. Therefore, the most interesting statistics, such as the skewness and the excess kurtosis, can be used for the purpose of testing whether the empirical distribution does have kurtosis and skewness just like a normal distribution. This undertaking has already been applied done with the Jarque-Bera test which rejected the null hypotheses, indicating that the sample distribution stems actually from a normal distribution set to a 1% significance level. To note, LB (24) statistics are employed for the take of testing the persistence of a high autocorrelation in the results’ first and second moments. The LB (24) statistics figured on Table 1 display just the existence of a high-order autocorrelation for Guinea, Sierra Leone, Liberia and Nigeria. These LB (24) statistics are supposed to be significant, reflecting the existence of a noticeable interdependence among second moments of returns. This also indicates that the returns’ heteroscedasticity should change with time. Such as result highlights well the use of estimation and variance of the autoregressive conditional heteroscedasticity (ARCH) model of [35]. As for the ARCH test, the null hypothesis of homoscedasticity is not accepted suggesting a further conditional time-varying in the number of death return dynamics.

### Long memory dependency and the Ebola virus

In a first place, the Robinson estimators of long memory parameters relevant to Table 2 reported periods, prove to be lower than 0.5 for the entirely series. Such a result highlights well that long memory dependency within the period turns out to be critically important. This might well be due to the recurrent shocks and breaks recently occurring in the African countries. A possible explanation for this lies in the fact that the invasive occurrences in the number of Ebola virus related deaths respective to Guinea, Sierra Leone, Liberia and Nigeria, during the studied period corresponding have lasted for extended periods and increased the long memory property as a mean process which responded asymmetrically and gradually to such shocks and breaks as already pointed out by [36].

**Table 2:** **Estimation of the long memory parameters number of deaths**

	**Guinea**	**Sierra Leone**	**Liberia**	**Nigeria**
GS	0.272	0.180	0.237	0.184
ARFIMA(1,d,1)	0.365(0.00)	0.604(0.00)	1.063(0.00)	0.122(0.17)
LW	0.751	0.725	1.065	0.651
2ELW	0.658	0.592	0.912	0.618
2ELWd	0.560	0.322	0.873	0.569

Notes: GS, LW, 2ELW and 2ELWd indicates respectively the Robinson, Local Whittle, 2 Stage Exact Local Whittle and Exact Local Whittle with detrending estimators. The value between (.) indicates the P-value.

**Figure 2:**
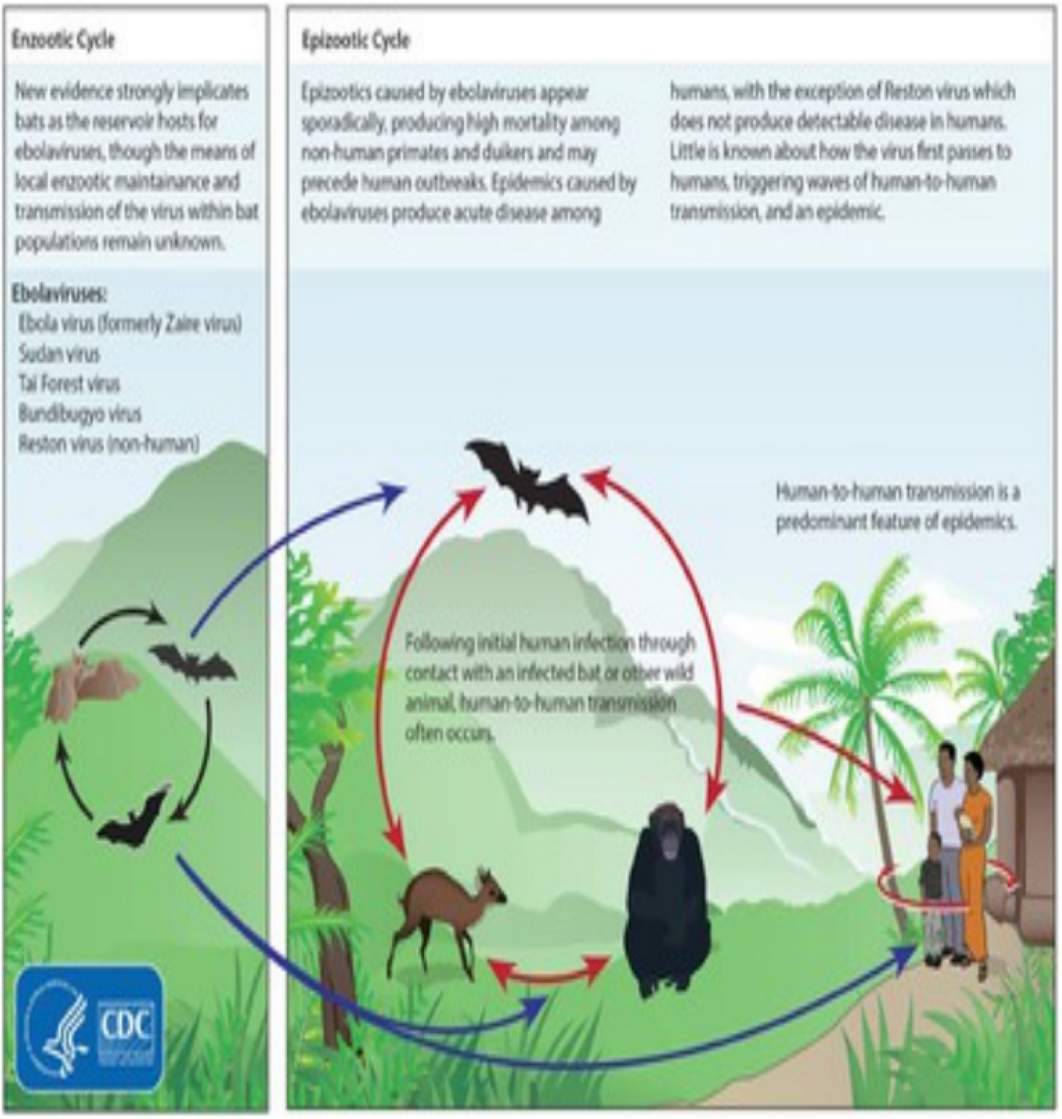
**Contagion of the Ebola virus between wildlife and human beings.**

**Figure 3:**
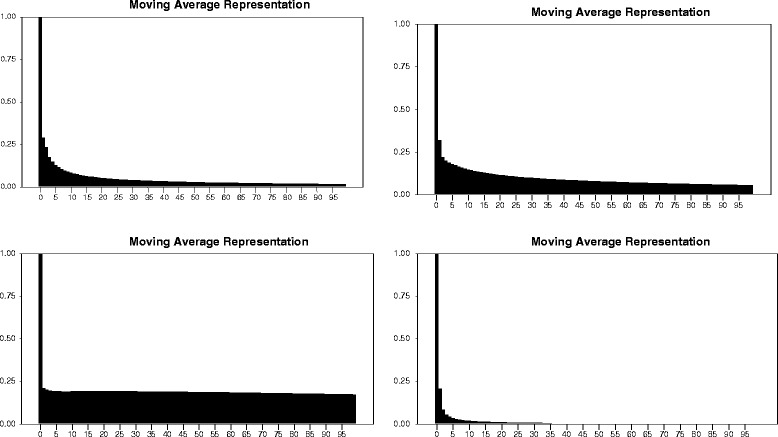
**Sample Autocorrelation Function for the daily returns of lags 0 to 95 and the 5% confidence level.**

**Figure 4:**
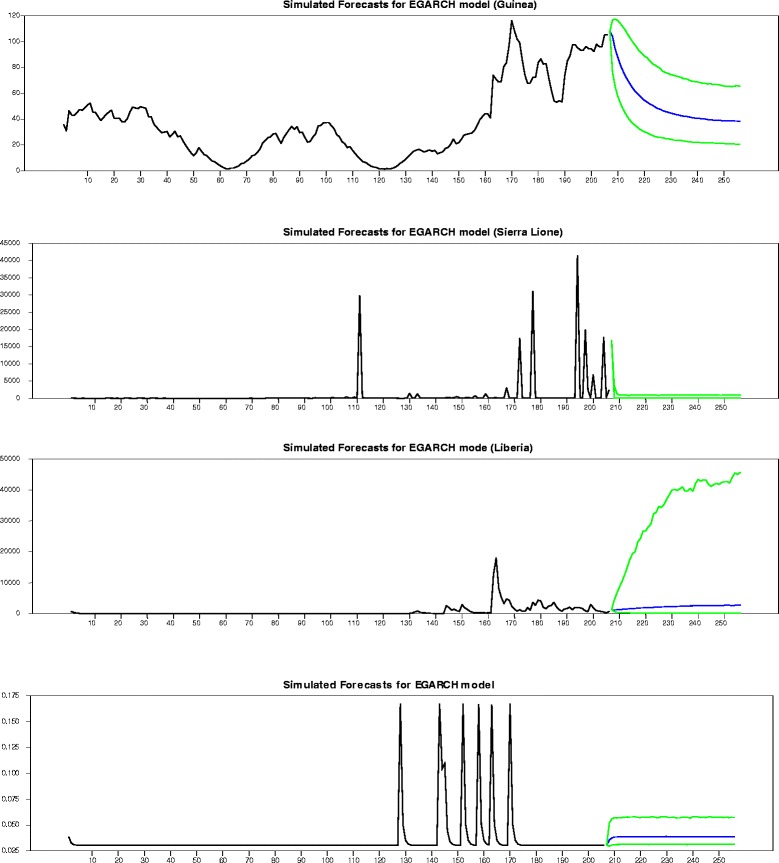
**Number of death daily index level from 2014-03-22 until 2014-12-30.** Indicate the number of death daily index level from 2014-03-22 until 2014-12-30. In total there are 260 observations. The red line represents the index level during the in-sample period and the blue graphics indicates where the out-of-sample period starts which is then represented by the black line.

The plot of Figure 2 depicts the Sample Partial Autocorrelation Function clearly highlighting the prevalence of a significant autocorrelation. The long memory process captures a very low frequency cycle in the number of Ebola virus incurred deaths, by permitting a slowly declining autocorrelation of a hyperbolic shape in the long horizon (see Figures 1 and 3 relevant to Guinea and Sierra Leone). Inversely, however, the short memory process is characterized by a fast exponential decline in autocorrelations as depicted by Figures 2 and 4 pertaining to Liberia and Nigeria. For the purpose of verifying the persistence of the Ebola virus, pertinent real shocks, we consider applying the LW, 2ELW and 2 ELW with trending.

Based on Table 2, the Robinson estimators (GS) do actually prove that $0\le \widehat{d}\le 0.5$. Indeed, this consists in a long-memory process case through still stationary, with a slow or smooth decay in the catching-up process. Concerning the ARFIMA(1,d,1) process, it has been demonstrated that $0\le \widehat{d}\le 0.5$ with respect to Guinea and Sierra Leone, underling a long memory and stationary process. In regard of both Liberian and Nigerian, cases, it has been prove that $\widehat{d}\ge 1$. In effect, this corresponds to an explosive to process case and a situation marked by where there is a strong magnification effect, with any initial difference being unexpected to be potentially reversed in the future.

In reality, this coincides with the “stochastic divergence” case liable to comparison with the initial deterministic divergence case. Regarding the 2ELW estimator, it has been demonstrated that $0.5\le \widehat{d}\le 1$, corresponding to a long memory process case, which is non-stationary through still reverting. In such a case, the process is featured with high persistence, whereby any distant past output difference would still have a long-lasting present inference. With respect to the 2ELWd estimator, it has also been demonstrated that $0.5\le \widehat{d}\le 1$ regarding the entirety of studied cases, except for Sierra Leone. What noting also, the number of deaths highest values (0.272 and 0.237, respectively for Guinea and Liberia), highlight well the persistent of shocks in the number of Ebola virus increased deaths and its contagion.

In fact, the Ebola virus can persist in the patient’s body for about 2 months and, the sick and dead people are contagious. Following the incubation period ranging between 2 and 21 days (a period with no symptoms and no contagious), the first symptoms^f^ begin to appear, and the affected person becomes contaminated on with the onset of the symptoms. The Ebola virus raised death occurs relatively quickly due to bleeding and unfortunately, there is no treatment or vaccine against the disease is yet available. This might well sound being plausible when the virus affects at fast countries within a small restricted geographic area, but spreads out to cover a wider geographical area and persists underway for several months (between 3 and 4 months). In addition, if the Ebola dynamics were to be modeled in real time, it would be critical to consider possible delays while report some cases and outcomes.

The Ebola virus transmission is marked by two major phases. The first started in July 2014, when Guinea, Liberia and Nigeria become affected with the shocks. As for, the second phase, it started in August 2014, when all the four countries become affected by at least a single shock during that month. Concerning Guinea and Nigeria, there were structural changes for two months, September (05/09, 24/09 and 26/09) and August (09/08, 16/08 and 27/08). This fact indicates an approximation of the Ebola virus quiet periods boundaries and times. This denotes that the number of fatal Ebola viruses appears to be is more dispersed during these two months owing to the increasing number of Ebola virus incurred deaths. As a matter of fact, 494 deaths were recorded in August reaching 739 deaths in late September in Guinea.

In Liberia, one might well note that the four points marking structural changes through the ICSS algorithm are sited during August and early September. During the end of the period, the number of deaths increased remarkably from 260 to 953. In regard of Nigeria, only two structural change points have been highlighted occurring between late August and early September. This might well has its explanation in the increase noticed in the number of deaths growing up from 1 to eight due to the Ebola virus. Unless it is imperative, the studied countries are recommended to suspend all their flights towards the countries where the Ebola haemorrhagic fever cases have proven to be remarkably serious, such as Guinea, Sierra Leone, Liberia and Nigeria. Indeed, for five months, the Ebola outbreak was the worst since the emergence of the initially high contagious hemorrhagic fever in 1976. Actually, it was at the origin of 1,145 reported deaths according to the World Health Organization (WHO), reporting 413 cases in Liberia, 380 in Guinea, Sierra Leone and 348 in Nigeria.

Such interesting findings appear to be unable to capture the asymmetry of volatility reaction to external shocks. For this reason, an EGARCH (1,1) model is estimated whose empirical results are reported in Table 3. Accordingly, the parameter γ_0_ is shown to be negative and significant except for Liberia and Nigeria. This may well further evidence in favor of variance reduction after a positive shock and vice versa.

**Table 3:** **Results of the EGARCH (1,1) models**

	**Guinea**	**Sierra Leone**	**Liberia**	**Nigeria**
*c*_1_	2.603	1.004	0.00	0.033
*δ*_0_	0.332***	2.812***	−0.156	−2.447***
*δ*_1_	0.921***	0.268***	0.930***	0.313***
*γ*_0_	−1.033***	1.095***	0.281***	−0.190***
*γ*_1_	0.260***	2.729***	−0.411***	0.035***

Notes: ***indicate the significance level at 10%.

Noteworthy, however, the estimated value of γ_0_ appears to differ greatly between (Guinea and Sierra Leone) on the one hand, and (Liberia and Nigeria) on the other hand. This suggests that the volatile countries’ exposure and liability to risks varies not only among (Guinea and Sierra Leone) and (Liberia and Nigeria), but also among the countries under review. Obviously, this might have an association with the number of deaths which seemed to be lower with respect to (Liberia and Nigeria) than to (Guinea and Sierra Leone), mainly on the onset of the Ebola virus disease. As for parameter δ_1_ which serves to measure the conditional volatility persistence, its estimators prove to be significant. The highest value related to this parameter has been recorded for Guinea and Liberia exceeding 0.9, in respect of its value registered in Sierra Leone and Nigeria. It also reveals volatility dynamics which appear to be more persistent with regard to Guinea. Besides, our results appear also to confirm the persistence of asymmetry in the volatility reaction to shock size. Indeed, the estimated value of γ_1_ is positive for all the countries except for Liberia, scoring its highest points in (Guinea and Sierra Leone), suggesting that the volatility dependency to market events is more important for both Guinea and Sierra Leone.

Regarding the number of deaths registered in Guinea, Sierra Leone Liberia and Nigeria, 260 daily data were pointed during the entire period from: 2014-03-22 until 2014-12-30. The in-sample period: from 2014-03-22 until 2014-10-14 consisted of 206 daily data points while the out-of-sample period from: 2011-01-03 until 2014-04-15 consisted of 54 daily data points. In Figure 2, the number of daily deaths recorded during the entire period is plotted. The in-sample period is plotted in black, and the green graphics indicate the lower graphics while and the upper graphics are highlights with the blue line.

A decrease in the number of Ebola virus incurred deaths was noticed during the following two months (November and December 2014). This is mainly due to a drastic reduction in the number of animals natural reservoirs of the virus. Indeed, the upper and lower figures appear to have a similar shape to the graph in blue. This indicates well a significant decrease in the number of the Ebola virus caused fatalities in Guinea. The virus transmission remains intense in Guinea with a clear exponential in the daily deaths number time series. In Sierra Leone, for instance, one may notice that the number of daily Ebola deaths remained constant during the following two months (November and December). This number marked some peaks from one day to another. As for Liberia, the number of the Ebola viruses is expected to increase over the next 60 days (November and December). Liberia contributed strongly in the high number of the whole outbreak cases registered during the last two months (November and December). Regarding, Nigeria, the number of forecast deaths registered via EGARCH process highlights this figure constancy over the coming months (November and December). For the sake of determining the causal relationship between Ebola and the studied countries, the Granger causality test is applied as introduced by [37].

### The Granger causality test

This part is interested to test the presence of Granger causality relationship in a bivariate model among different series (Guinea, Sierra Leone Liberia and Nigeria) on the studied countries. To note, the Granger causality test is a statistical hypothesis test helpful in determining whether a certain time series is useful for forecasting another (see [7]). Ordinarily, regressions help reflect correlations, but Granger argued that causality in economics could well be reflected by measuring the ability of predicting a certain time series’ future values using another series’ past value. Since the “true causality” issue is deeply philosophical, econometricians assert that the Granger test finds only “predictive causality” [38].

In this section, the Granger causality tests are going to be administered, using. We use the unrestricted model through which causality will be investigated between the number of deaths respective to Guinea, Sierra Leone, Liberia and Nigeria. The relevant results are summarized in Table 4, below. The number occurring in each cell of the probability columns indicates the presence of a significant causal relationship among the four African countries under consideration. A superscript (*, **, ***) reflects the F-test statistical significance for Granger causality at 10%, 5% and 1% significance levels respectively. The results indicate three patterns:

**Table 4:** **Results of Granger causality tests**

**Null hypothesis**	**F-statistic**	**Probability**
Guinea does not Granger Cause Sierra Leone	1.206	0.27
Guinea does not Granger Cause Liberia	**4.049**	0.04*
Guinea does not Granger Cause Nigeria	0.307	0.579
Sierra Leone does not Granger Cause Guinea	1.485	0.224
Sierra Leone does not Granger Cause Liberia	**2.984**	0.08**
Sierra Leone does not Granger Cause Nigeria	0.032	0.857
Liberia does not Granger Cause Guinea	**3.913**	0.04**
Liberia does not Granger Cause Sierra Leone	**13.408**	0.000***
Liberia does not Granger Cause Nigeria	0.206	0.650
Nigeria does not Granger Cause Guinea	0.697	0.404
Nigeria does not Granger Cause Sierra Leon	0.004	0.946
Nigeria does not Granger Cause Liberia	0.008	0.926

- *Group 1*: it is composed of four countries; Guinea^g^ is the origin source of Ebola virus, i.e. the African countries are recently affected with the Ebola virus through Guinea. This group includes Guinea, Sierra Leone, Liberia and Nigeria.
- *Group2:* is composed of such countries as Guinea, Liberia and Nigeria which are affected by Sierra Leone^h^ where a high number of deaths predominate.
- *Group 3:* consists of such countries high as Guinea, Sierra Leone and Nigeria affected by the Ebola virus as transmitted from Liberia^i^.
- *Group 4:* includes Guinea, Sierra Leone and Liberia in which the virus is transmuted from Nigeria^j^.

The number of causal relations hips is depicted in the following table. A causal relationship is persistent in each of the first three groups, but no relationship has been noticed to exist in the fourth group.

According to Table 4 pertaining to the Granger causality test, the disease appears to be transmitted from Guinea to Liberia (Fisher 4049 is calculated at a 5% significance) threshold. This implies that there is a high probability for the disease to have been spread from Guinea to Liberia.

Moreover, it is clear that the disease in Sierra Leone has resulted in the same disease in Liberia (Fisher calculated is 2.984 with a probability of 0.08). Accordingly, one might well infer that the Ebola virus may have been spread from Guinea through Sierra Leone to Liberia. It has also been shown that the Ebola virus has been spread from Liberia to Sierra Leone. These two findings several that the Ebola virus native countries are discovered to be Guinea and Sierra Leone. For the purpose checking the right transmission of the Ebola virus among these countries, we have made appeal to the DCC-MGARCH technique. In fact, this framework enables us to estimate two main parameters, the first of which helps determine the persistence of the Ebola virus, while the second serves to demonstrate the Ebola virus shock contamination effect through the considered countries.

The two major main parameters considered are *a*_*m*_ and *b*_*n*_, respectively, underlining the persistence of shock effect and contamination. Based on Table 5, one can notice that coefficient *a*_*m*_ proves to be high and significant (0.583) highlighting the continuity of the Ebola virus impact. Consequently, a high probability remains as to the virus transmission from Guinea to Nigeria. As matter of fact, coefficient *a*_*m*_ has been demonstrated and deemed important because the study period has covered only 8 months (March-October 2014). During this fairly short period, several structural changes have been noticed to take place (5 in Guinea; 4 in Sierra Leone and Liberia and 2 in Nigeria) as figured in Table 6. This implies well that these dates help indicate structural changes of the peak rather than the Ebola virus shock persistence of in the surveyed countries. Regarding the second parameter, *b*_*n*_, the Ebola virus spread transmission is observed to occur between Guinea-Liberia, Guinea-Sierra Leone and Sierra Leone-Liberia. Yet, parameter *b*_*n*_ appears to be not significant owing mainly to of the lack of data (only 206 observations) and the GARCH family. Indeed, the latter entails the availability of a large amount of data, parameters’ cruciality. For this reason, we have reckoned it more useful to apply the DCC-MGARCH rather than the BEKK-MGARCH which admits further parameters than does the DCC version. As results, we consider it’s rather effective to study the Ebola virus contagion via DCC figures.

**Table 5:** **Results of the MGARCH-DCC (1,1) models**

	**Guinea-Liberia**	**Guinea-Nigeria**	**Guinea-Sierra Leone**	**Sierra Leone-Liberia**
*c*_1_	5.570(0.26)	0.102(0.05)	6.56(0.27)	0.397(0.94)
*c*_2_	2.95(0.00)	2.62(0.11)	1.23(0.42)	0.969(0.87)
*w*_1_	1539(0.29)	0.020(0.70)	224.21(0.69)	908.26(0.43)
*w*_2_	44.14(0.15)	50.88(0.18)	60.082(0.14)	248.02(0.63)
*α*_1_	0.201(0.00)	0.12(0.00)	0.065(0.00)	0.052(0.03)
*α*_2_	0.170(0.00)	0.097(0.00)	0.065(0.00)	0.047(0.24)
*β*_1_	−0.361(0.260)	0.676(0.15)	0.608(0.42)	0.36(0.64)
*β*_2_	0.430(0.123)	0.453(0.18)	0.41(0.25)	0.61(0.36)
*α*_*m*_	0.078(0.58)	0.583(0.03)	0.17(0.74)	0.09(0.71)
*b*_*n*_	0.310(0.32)	0.008(0.98)	0.24(0.80)	0.469(0.87)

Notes: The value between (.) is P-value.

**Table 6:** **The structural breaks and their emergence dates**

	**Guinea**	**Sierra Leone**	**Liberia**	**Nigeria**
1	23-03	07-07	09-08	24-08
2	29-08	08-07	16-08	05-09
3	05-09	01-09	27-08	-
4	24-09	01-10	03-09	-
5	26-09	-	-	-

**Figure 5:**
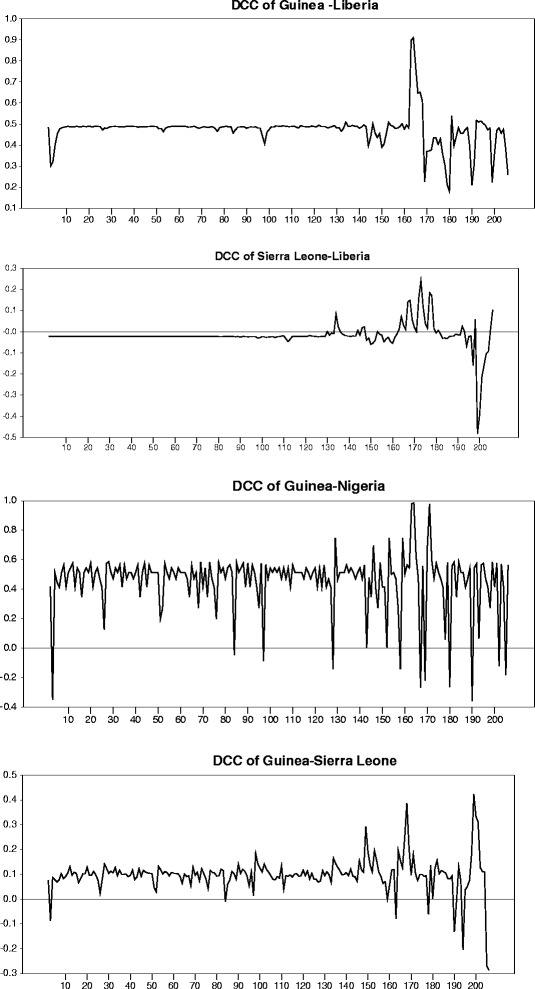
**Dynamic Correlation Coefficients of returns between countries.**

Figure 5 depicts the Dynamic Correlation Coefficients respective to four country pairs. The correlation variation among each pair can be well observed via DCC coefficients. Thus once positive and close to 1, correlation is discovered to indicate a similar trend in the number of deaths. However correlation proves to be negative with an absolute value close to 1, it would then indicate an opposite trend in the number of deaths. Actually, DCC has been discovered to be comprised between 0.4 and 0.6 during the first four months (March-June) for both pairs Guinea-Nigeria as well as Guinea-Liberia, indicating the Ebola virus spread between Guinea and Nigeria. In regard of the other two pairs, contagion seems less prevalent in both Guinea-Sierra Leone and Sierra Leone-Liberia and has been proven to present exclusively during the last month (with DCC comprised between 0.3 and 0.5). So, it can be concluded that Guinea appears to be the source origin of contagion, as one may notice the Ebola virus shock spread from Guinea to Sierra Leone to Liberia. Indeed, on March 31, the WHO did confirm the virus spread to Liberia. On April the 17th, the number of deaths ranged between 131 and 209 in both countries.

On August 26, the African Development Bank estimated that Ebola outbreak could cost between 1 and 1.5 percentage points of the GDP in Liberia, Sierra Leone and Guinea, starting it a “country which began to recover difficult years of crisis, civil wars of the 60s, 80s and 90s”^k^.

## Discussion

On September 17th, a report released from the World Bank warned against a “catastrophic” economic impact the epidemic may have on Liberia, Sierra Leone and Guinea; due to the virus associated “fear factor” paralyze activities. According to the World Bank orientations, the combined GDPs of Liberia, Guinea and Sierra Leone could be cut by $ 359 million in 2014 and $ 809 million by 2015 if the epidemic were to remain uncontained. As a result, economic growth would then fall, in the following year, from 11.7 points to 8.9 points in Liberia and Sierra Leone, with a risk of plunging the two poorest countries in recession^l^.

Criticized in an internal report for the delay to take the necessary measure relevant to curb Ebola outbreak, the World Health Organization announced that the two anti-Ebola vaccines would be tested as soon as possible in Switzerland, subject to authorization of the medical authorities. French researchers beginning to the Commission for Atomic Energy and Alternative Energies (CEA) have also announced that a rapid test has been developed to diagnose the virus within fifteen minutes. Since its introduction in early March, the virus has been responsible for killing more than 4,500 people, mainly in Liberia, Sierra Leone and Guinea, according to the latest WHO released report (October 2014).

## Conclusions

Throughout, the scone of this study paper attempts have been made to test whether the Ebola virus can stand as an origin source of contagion during 2014 transmission process using the number of deaths data available from the World Health Organization. More particularly, we have undertaken to examine whether the Ebola virus can lead to contagious effects implicated on both conditional means and volatilities of its relevant number of deaths occurring during the recent period (March-October 2014). In fact previous studies have failed to take account of the important distinction both of the interdependence and contagion concepts. In this paper, contagion is defined as being the significant spillover of the asset-specific idiosyncratic virus during the crisis and as well following the first affection by the Ebola virus [23]. For an effective control of the economic fundamentals, we have considered relying on an international number of death models, which have provided a theoretical basic background in selecting the pertinent health fundamentals.

The empirical findings have revealed that contagion-in-mean effects turn out to be multidirectional given the fact that the Ebola virus chocks emanating from any of the four studied countries can sweep across throughout the entirety of investigated countries; however, contagion-in-volatility effects appear to be driven mainly by the Ebola epidemic stemming negative return shocks. This empirical result indicates well that shocks to countries’ return can eventually become contagious not only at the volatility level, but also at the mean level, significantly implying that the number of deaths communication can actually constitute a major cause of contagion throughout the recent period.

## Endnotes

^a^The virus family of which Ebola is a member, “Filoviruses” are far more ancient than previously thought.

^b^Ebola-Reston virus was recognized in monkey export ability in the Philippines. No human virus was identified.

^c^Specifically, contagion is defined in this paper as being significant spillovers of asset-specific idiosyncratic shocks during the crisis, after the Ebola virus or systematic risks have been accounted for [39].

^d^Weekly data are used here to get meaningful statistical generalizations and obtain a better picture of the movements of Ebola virus cases’ number.

^e^As a first step, stationarity in the time series is checked by applying the Augmented Dickey Fuller (ADF) test. The results allow us to reject the null hypothesis stipulating that the Ebola virus returns have a unit root in favour of the alternative hypothesis (even at 5% critical value).

^f^Fever, weakness, muscle and joint pain, diarrhea, internal and external bleeding.

^g^In Guinea, there are 1298 cases, of which 1062 are confirmed, with 768 deaths. The epidemic epicenter is Guéckédou, Guinea, but other affected provinces include also’: Beyla Conakry, Coyah, Dabola Dalaba, Forecariah Macenta Nzérékoré Kindia Kérouané, Kissidougou Dubréka and Lola Yomou. The conakry isolation center was set up by MSF. It seems that appearence of new cases in the Guéckédfou region is decreasing (World Health Organization).

^h^In Sierra Leone, there are 2948 cases with 880 deaths, among 2596 confirmed cases. All regions are discovered to be affected. The isolation and diagnosis center is sited in Kenema. The MSF has also implemented an isolation Kailahun and Lakka structures (Lakka Hospital) on the outskirts of Freetown. The Choitrams Hospital can no longer accept hospitalization.

^i^In Liberia, there are 3924 cases in total, 941 of which were confirmed, in 1795 with 1188 and 2210 suspicious deaths. It is likely that these figures are far below reality. The Diagnostic Center is located in the Liberian Institute of Biomedical Research (LIBR) near Monrovia. The hospitals fully devoted to the Ebola case are: ELWA, ELWA3, JFK in Monrovia, are hospitals which cannot cope with the influx of new patients.

^j^In Nigeria, the last new case dates back to September 5 in Lagos and Port Harcourt September 1 (15 in Lagos and 4 in Port Harcourt). The epidemic appears under control in Nigeria but there are still 25 subject contacts under observation. The US CDC reduced its recommendation travel Level 1 (can proceed with caution).

^k^Ebola could cost “a lot” to the African economy, Le Monde, August 26, 2014.

^l^Ebola: an economic impact “catastrophic” envisaged by the World Bank, Le Monde, 17 October 2014.
